# Implications and prognostic impact of mass spectrometry in patients with newly-diagnosed multiple myeloma

**DOI:** 10.1038/s41408-022-00772-9

**Published:** 2023-01-04

**Authors:** Elias K. Mai, Stefanie Huhn, Kaya Miah, Alexandra M. Poos, Christof Scheid, Katja C. Weisel, Uta Bertsch, Markus Munder, Oscar Berlanga, Dirk Hose, Anja Seckinger, Anna Jauch, Igor W. Blau, Mathias Hänel, Hans J. Salwender, Axel Benner, Marc S. Raab, Hartmut Goldschmidt, Niels Weinhold

**Affiliations:** 1 Department of Internal Medicine V, Heidelberg University Hospital, Heidelberg, Germany; 2 National Center for Tumor Diseases (NCT) Heidelberg, Heidelberg, Germany; 3 Division of Biostatistics, German Cancer Research Center (DKFZ), Heidelberg, Germany; 4 Department of Internal Medicine I, University Hospital Cologne, Cologne, Germany; 5 Department of Oncology, Hematology and Bone Marrow Transplantation with Section of Pneumology, University Medical Center Hamburg-Eppendorf, Hamburg, Germany; 6 Department of Internal Medicine III, University Medical Center Mainz, Mainz, Germany; 7 The Binding Site Group Ltd, Birmingham, United Kingdom; 8 Department of Hematology and Immunology, Myeloma Center Brussels, Vrije Universiteit Brussel, Brussels, Belgium; 9 Institute of Human Genetics, University of Heidelberg, Heidelberg, Germany; 10 Medical Clinic, Charité University Medicine Berlin, Berlin, Germany; 11 Department of Internal Medicine III, Klinikum Chemnitz, Chemnitz, Germany; 12 Asklepios Tumorzentrum Hamburg, AK Altona and AK St. Georg, Hamburg, Germany

**Keywords:** Myeloma, Myeloma, Risk factors, Translational research, Genetic testing

## Abstract

Mass spectrometry (MS) is a promising tool for monitoring monoclonal protein in plasma cell dyscrasias. We included 480 transplant-eligible newly-diagnosed multiple myeloma (MM) patients from the GMMG-MM5 trial (EudraCT No. 2010-019173-16) and performed a retrospective MS analysis at baseline (480 patients) and at the pre-defined, consecutive time points after induction (444 patients), prior to maintenance (305 patients) and after one year of maintenance (227 patients). We found that MS negativity was significantly associated with improved progression-free survival (PFS) even in patients with complete response (CR) at all investigated follow-up time points. The prognostic impact was independent of established risk factors, such as the revised International Staging System. Combining MS and baseline cytogenetics improved the prediction of outcome: MS-positive patients with high-risk cytogenetics had a dismal PFS of 1.9 years (95% confidence interval [CI]: 1.6–2.3 years) from the start of maintenance. Testing the value of sequential MS prior to and after one year of maintenance, patients converting from MS positivity to negativity had an excellent PFS (median not reached) while patients converting from MS negativity to positivity progressed early (median 0.6 years, 95% CI: 0.3-not reached). Among patients with sustained MS positivity, the baseline high-risk cytogenetic status had a significant impact and defined a group with poor PFS. Combining minimal residual disease (MRD) in the bone marrow and MS allowed the identification of double negative patients with a favorable PFS (median 3.33 years, 95% CI: 3.08-not reached) and no overall survival events. Our study provides strong evidence that MS is superior to conventional response monitoring, highlighting the potential of MS to become a new standard. Our data indicate that MS should be performed sequentially and combined with baseline disease features and MRD to improve its clinical value.

Clinical Trials Register: EudraCT No. 2010-019173-16

## Introduction

Novel therapeutics have significantly improved response rates as well as the depth of response in patients with multiple myeloma (MM) [1]. However, despite complete responses (CR) patients stratify into those who achieve long-term remission and those who relapse within a few months [2]. Hence, there is a high need for techniques that track residual disease with increased sensitivity and specificity as compared to the conventional response criteria [3]. Molecular techniques, such as next-generation flow cytometry or next-generation sequencing, fulfill both criteria but come with drawbacks. They require inconvenient and painful procedures to obtain bone marrow samples and do not account for the potential inhomogeneous distribution of residual disease (e.g. focal intramedullary or extramedullary disease), since they are based on a sample from a randomly selected site at the iliac crest [4].

The minimally invasive technology mass spectrometry (MS), which is amenable to automation, is emerging as a promising approach for detecting and monitoring monoclonal proteins in the peripheral blood (PB) [5–9]. MS has been shown to be superior to standard electrophoretic methods for the detection of monoclonal immunoglobulins, such as serum immunofixation (IFE) [10–13]. Furthermore, recent data suggests a role for MS as a complementary approach for the detection of minimal residual disease (MRD) [11, 13], overcoming the limitations of bone marrow-based methods for identifying systemic disease.

To provide further evidence of the clinical value of MS and to explore whether it provides independent prognostic information, we have retrospectively tracked treatment response by serum MS in patients who had been enrolled in the German-speaking Myeloma Multicenter Group (GMMG) multicenter phase III GMMG-MM5 trial (EudraCT No. 2010-019173-16). The primary endpoint of the trial investigated continuation versus omission of lenalidomide maintenance for patients achieving a CR [14], which allowed us to compare the prognostic impact of MS in CR patients with or without maintenance. Other strengths of this study include 1) a rather homogeneous treatment with a proteasome-inhibitor containing induction therapy followed by high-dose melphalan (HDM) and autologous stem-cell transplantation (ASCT) and lenalidomide consolidation/maintenance, 2) the availability of a baseline sample in all patients, which allowed us to determine the unique mass of the monoclonal protein for tracking, and 3) the availability of comprehensive clinical and cytogenetic data.

## Patients and methods

### Study design and participants

For quantitative immunoprecipitation mass spectrometry (QIP-MS), we included peripheral blood (PB) serum samples from 480 GMMG-MM5 patients at baseline, from 444 of these patients after three cycles of either VCD or PAd induction therapy and prior to HDM/ASCT, from 305 patients prior to maintenance treatment or observation in case of CR in arm B of the trial, and from 227 patients after one year (±3 months) of maintenance treatment or observation. The design and main outcomes of the prospective, open-label, multicenter phase III trial GMMG-MM5 trial, which enrolled a total of 604 transplant-eligible patients with newly-diagnosed MM, have been previously reported [14]. The study design is also shown in Supplementary Fig. 1. Response within the trial was assessed according to the International Myeloma Working Group (IMWG) criteria as described [14, 15]. Response assessment was performed using serum electrophoresis (SPEP) and IFE in the serum or urine to quantify and detect the monoclonal protein. For CR assessment, a bone marrow puncture was mandatory and less than 5% plasma cells (by cytology or histology) in the bone marrow were required. Yet, bone marrow punctures were not mandatory for patients in arm A. All patients provided written informed consent. The trial was approved by the ethics committee of the University of Heidelberg and all participating sites and was conducted according to the European Clinical Trial Directive and the Declaration of Helsinki.

### Mass spectrometry for detection of monoclonal immunoglobulins

QIP-MS was carried out using the automated EXENT^®^ assays and system (The Binding Site Group Ltd., UK; assays and system in development). Briefly, sheep polyclonal antibodies (anti-IgG, -IgA, -IgM, -total κ, -total λ, free κ and free λ) covalently attached to paramagnetic microparticles were separately incubated with serum to enrich for immunoglobulins. The microparticles were washed and treated to simultaneously elute and reduce patient immunoglobulins into their constituent heavy and light chains. Light chain mass spectra were acquired by matrix-assisted laser desorption ionization time-of-flight MS (MALDI-TOF MS). Readout and interpretation were performed using proprietary software to yield immunoglobulin isotype and mass-to-charge ratio (*m/z*). The patient’s specific molecular mass of the monoclonal light chain was defined at baseline and was used to track the presence of the monoclonal protein during follow-up. A positive score by mass spectrometry in follow-up samples was based on the presence of a monoclonal protein at the same m/z (+/− 10 for the doubly charged light chain) as determined at baseline. Two experienced analytical scientists blinded to all clinical information reported on the MS spectra.

### Risk stratification by fluorescence in situ hybridization and the revised International Staging System

Interphase fluorescence in situ hybridization (FISH) analysis was performed as described previously [16]. High-risk cytogenetics (clone size ≥10%) were defined: (1) either deletion 17p13 and/or translocation t(4;14) and/or translocation t(14;16) according to IMWG consensus [16, 17], as well as gain(1q21) (>2 copies), which is an independent prognostic marker [18]. The Revised International Staging System (R-ISS) was calculated as described [19].

### Allele-specific oligonucleotide polymerase chain reaction for detection of minimal residual disease

For MRD analyses, DNA was extracted after density gradient separation of lymphocytes from bone marrow (BM) aspirates, which was then stored at −20 °C until analysis. We used patient-specific quantitative allele-specific oligonucleotide PCR (qASO-PCR) assays on immunoglobulin heavy chain (IgH) and kappa/lambda (k/λ) light chain as recently described [20, 21]. For MRD-negative results, a minimum of 10^6^ cell equivalents had to be tested without any positive amplification if this amount of material was available to reach a sensitivity for MRD negativity of at least 1 × 10^−6^.

### Statistical design and analysis

The Kaplan-Meier method was used for survival analyses. PFS time was measured from the respective landmark to relapse or death from any cause, whichever occurred first. OS was defined as time from the respective landmark until death from any cause. For analyses of sustained MS from start of maintenance/observation until one year, PFS and OS were calculated from the second time point (after one year) and only patients who did not have a prior progression event were included. MS test results were evaluated in a multivariable Cox regression model including established risk factors. Main analyses were undertaken using R (v4.0.4) software.

## Results

### Mass spectrometry results are associated with outcome at multiple time points

**Fig. 1:**
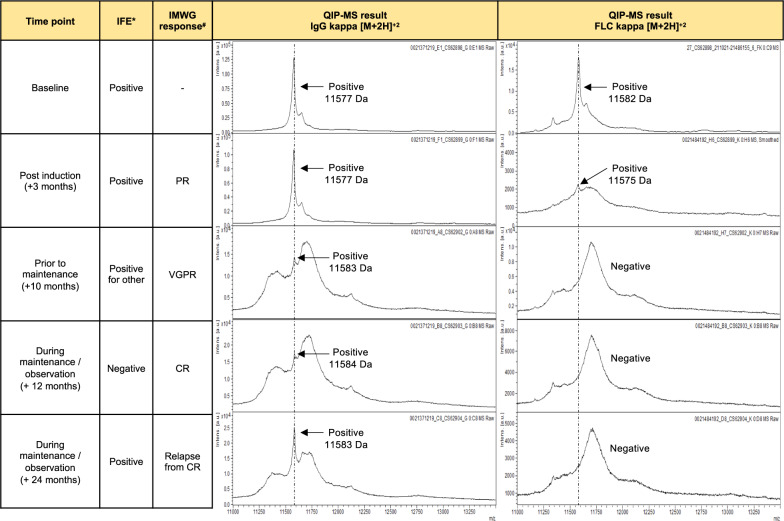
Example on longitudinal response monitoring using QIP-MS and immunofixation. Exemplary case showing the immunofixation (IFE) results, IMWG response and QIP-MS spectra for the monoclonal intact immunoglobulin and free light chain at selected time points. In this patient, QIP-MS also identified a monoclonal IgG kappa (*m/z* = 11577) + free kappa (*m/z* = 11582) at baseline, indicating the presence of a patient-specific clone co-expressing monoclonal intact immunoglobulin and free light chain. During maintenance/observation (after 24 months) the patient achieved a complete response (CR) while remaining QIP-MS positive. * serum IFE # according to IMWG criteria [15], IFE immunofixation, IMWG International Myeloma Working Group, PR partial response, VGPR very good partial response, CR complete response.

We used the QIP-MS for longitudinal monitoring of 480 patients with PB serum samples at baseline and at least one additional time point. Combining MS for intact immunoglobulins and free light chains, a monoclonal immunoglobulin could be identified at baseline for each patient, which allowed us to longitudinally track the respective tumor clone in all patients. An example is shown in Fig. 1. Baseline characteristics of the cohort are presented in Table 1. Median follow-up of the cohort was 57 months for both PFS (interquartile range: 49–64 months) and OS (interquartile range: 50–66 months) with 296 PFS and 131 OS events, respectively.

**Table 1:** Baseline characteristics of the analyzed cohort within the GMMG-MM5 trial.

Variable^a^	Subcategory	Value *n* (%)^b^
Age	years [median (range)]	59 (32–70)
Gender	male	284 (59.2)
	female	196 (40.8)
Study arm	A1	116 (24.2)
	A2	121 (25.2)
	B1	114 (23.8)
	B2	129 (26.9)
Immunoglobulin type	IgG kappa	208 (43.3)
	IgG lambda	79 (16.5)
	IgA kappa	63 (13.1)
	IgA lambda	39 (8.1)
	Bence Jones kappa	59 (12.3)
	Bence Jones lambda	27 (5.6)
	IgD lambda	5 (1.0)
R-ISS	I	128 (26.7)
	II	261 (54.4)
	III	65 (13.5)
	missing	26 (5.4)
High-risk cytogenetics	no	206 (43.9)
	yes	222 (46.3)
	missing	52 (10.8)
del(17p)	no	380 (79.2)
	yes	55 (11.5)
	missing	45 (9.4)
t(4;14)	no	383 (79.8)
	yes	48 (10.0)
	missing	49 (10.2)
t(14;16)	no	411 (85.6)
	yes	12 (2.5)
	missing	57 (11.9)
gain(1q21)	no	260 (54.2)
	yes	174 (36.3)
	missing	46 (9.6)

^a^The analysis included 480 (79.5%) of 604 patients randomized within the GMMG-MM5 trial with an MS sample at baseline and at least one additional time point (post induction, prior to maintenance therapy/observation or after 1 year of maintenance treatment/observation).

^b^Unless otherwise indicated.

**Fig. 2:**
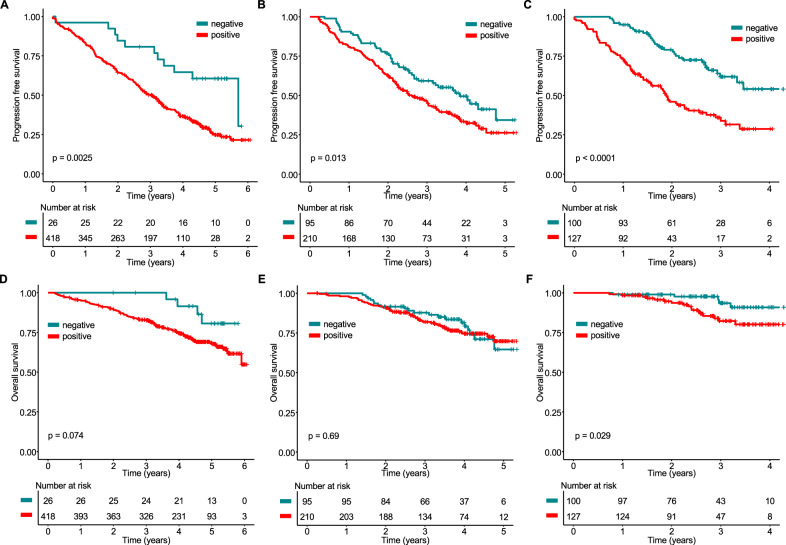
Prognostic value of mass spectrometry at defined time points. Progression-free (PFS) and overall survival (OS) of GMMG-MM5 patients stratified by the mass spectrometry test result (negative/positive) after induction therapy (**A** + **B**), prior to maintenance therapy/observation (**C** + **D**) and after one year of maintenance treatment/observation (**E** + **F**). PFS and OS times were measured from the respective landmarks.

After three cycles of induction therapy with PAd (Bortezomib/Doxorubicine/Dexamethasone) or VCD (Bortezomib/Cyclophosphamide/Dexamethasone), a negative MS test result was infrequent (6%, 26/444). Nevertheless, in a landmark analysis from the end of induction, it defined a group with significantly improved PFS (hazard ratio [HR] = 0.41; 95% confidence interval [95% CI]: 0.22–0.74; *p* = 0.003) and a trend was seen towards better OS (HR = 0.41 95% CI: 0.15–1.12, *p* = 0.08, Fig. 2A, B). Prior to maintenance/observation, the proportion of MS negative tests increased to 31% (95/305 patients). Again, a negative MS test was associated with improved PFS (HR = 0.66, 95% CI: 0.47–0.92, *p* = 0.01) but there was no significant difference for OS (HR = 0.90, 95% CI: 0.53–1.52, *p* = 0.7, Fig. 2C, D). Compared to these two time points, the strongest prognostic value of single time point MS testing was seen after one year of maintenance/observation: 44% (100/227) of patients had a negative MS test and experienced superior PFS (HR = 0.37, 95% CI: 0.25–0.57, *p* < 0.001) and OS (HR = 0.35, 95% CI: 0.13–0.94, *p* = 0.04, Fig. 2E, F).

A recent study demonstrated that the extended half-life of IgG could impact disease monitoring by MS [22]. In line, only 2% of IgG but 18% of Bence Jones (BJ) MM patients were MS negative after induction in our study (*p* < 0.001, Supplementary Table 1). A significant difference between these two groups (24% vs 54%, *p* < 0.001) was also seen before maintenance/observation. To account for the extended half-life of IgG, Abeykoon et al. proposed to perform MS at least 6 months from ASCT or thereafter. In support of this notion, there was no significant difference in MS negativity between IgG and BJ MM patients (40% vs. 53%, *p* = 0.2) after one year of maintenance/observation in our study, which contributes to the strong prognostic value of single MS testing at this later time point.

### The mass spectrometry test result constitutes an independent prognostic factor

**Fig. 3:**
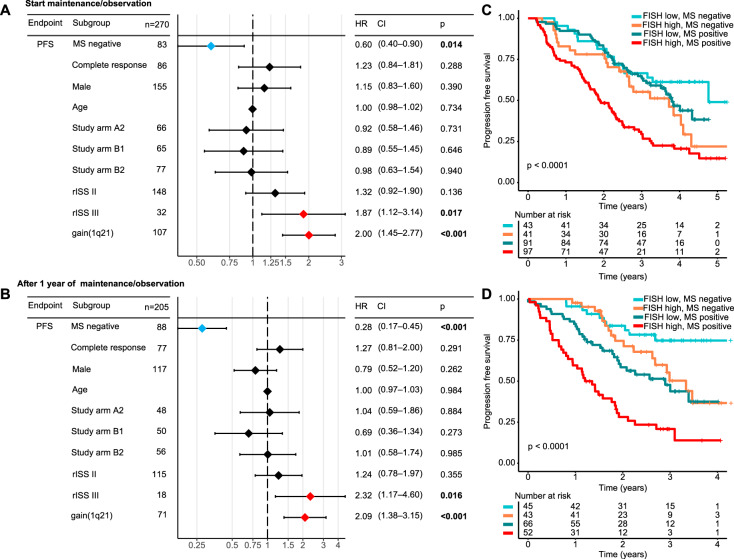
Independent prognostic impact of mass spectrometry and its combination with established high-risk markers. Results of a multivariable model for PFS from the start of maintenance therapy/observation (**A**), and after one year (±3 months) of maintenance/observation (**B**). PFS from start of maintenance therapy (**C**) and after one year of maintenance/observation (**D**) stratified by the combination of mass spectrometry (MS) and baseline high-risk cytogenetics. High-risk cytogenetics were defined by the presence of t(4;14), t(14;16), del(17p) and/or gain(1q21). Study arms: A1 = PAd induction therapy and lenalidomide maintenance independent on response status; A2 = VCd induction therapy and lenalidomide maintenance independent on response status; B1 = PAd induction therapy and lenalidomide maintenance if less than complete response or observation in case of complete response; B2 = VCd induction therapy and lenalidomide maintenance if less than complete response or observation in case of complete response. FISH flourescence in situ hybridization, MS mass spectrometry, rISS revised International Staging System.

Next, we evaluated the prognostic value of MS testing at the three defined time points in a multivariable model, which included age at diagnosis, gender, treatment arm, R-ISS stages and gain(1q21) status (Fig. 3A, B). Furthermore, we included conventional response (CR vs. no CR), with CR being defined as <5% plasma cells in the bone marrow and absence of the monoclonal protein in the serum and urine according to SPEP and IFE. We focused on PFS and the two time points prior to and after one year of maintenance/observation. For completeness OS results for these time points are shown in Suppl. Fig. 2A, B. The MS test result was an independent prognostic factor for PFS (prior to maintenance therapy: HR = 0.60, 95% CI: 0.40–0.90, *p* = 0.01; and after 1 year of maintenance therapy/observation: HR = 0.28, 95% CI: 0.17–0.45, *p* < 0.001). Other significant prognostic factors for PFS were R-ISS stage III and gain(1q21).

### Mass spectrometry improves the prognostic value of established risk markers

The prognostic value of the depth of response, as assessed by conventional response, MRD in the BM or functional imaging, is impacted by baseline disease features, such as high-risk cytogenetics [23, 24]. The results of our multivariable survival model suggest that the same holds true for response assessment by MS. Indeed, when combining the FISH high-risk markers of the R-ISS del(17p13), t(4;14), and t(14;16) as well as gain(1q21) at baseline with MS test results before maintenance/observation, patient groups with excellent or dismal outcomes could be defined (Fig. 3C). The best outcome was seen for patients without high-risk cytogenetics and MS negativity (median PFS: 4.8 years, 95% CI: 3.3—not reached), while patients with high-risk FISH and MS positivity had a median PFS of just 1.9 years (95% CI: 1.6–2.3 years) from the start of maintenance/observation. Highlighting the value of combining molecular and response data, patients with high-risk cytogenetics but a negative MS test had a significantly better outcome compared to high-risk patients with MS positivity (HR = 0.54, 95% CI: 0.34–0.87, *p* = 0.01). As shown in Fig. 3D, separation was even more pronounced when combining MS test results and cytogenetic risk status after one year of maintenance/observation. The best PFS outcome was again observed for patients without high-risk cytogenetics and MS negativity (median PFS: not reached), while MS testing was able to significantly discriminate favorable vs. dismal outcome in patients with high-risk cytogenetics (median PFS; MS negative: 3.3 years, 95% CI: 2.6—not reached vs. MS positive: 1.3 years, 95% CI: 0.9–1.9; HR = 0.29, 95% CI: 0.16–0.52, *p* < 0.001). OS results for the two time points are shown in Supplementary Fig. 2C, D.

### Mass spectrometry in patients with complete response and the impact of lenalidomide maintenance

**Fig. 4:**
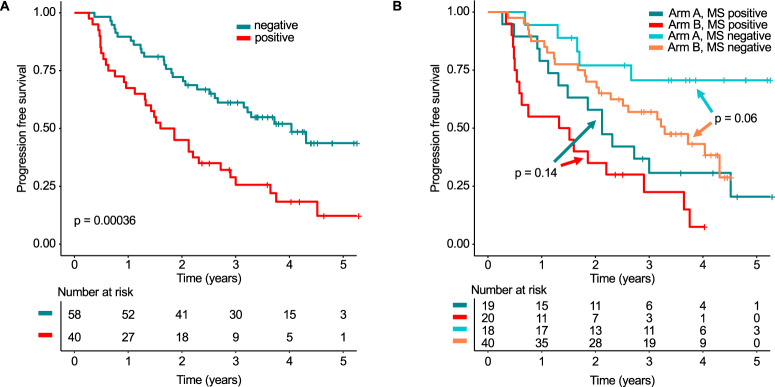
Prognostic Impact of mass spectrometry in patients with complete response. **A** PFS landmark analysis of patients in CR from the start of maintenance therapy/observation stratified by mass spectrometry (MS). In (**B**) patients in CR were stratified by the combination of MS and the GMMG-MM5 treatment arm (arm A lenalidomide maintenance for a maximum of 2 years, arm B observation only, if CR).

We recently showed that omitting maintenance in CR patients resulted in significantly worse outcomes [14], suggesting a high proportion of CR patients with significant residual disease requiring continued treatment. In line with this observation, we ascertained MS positivity in 41% (40/98) of CR patients prior to maintenance/observation, and in a landmark analysis from start of maintenance these patients had a median PFS of just 1.7 years (95% CI: 1.3–2.9 years) compared to 4.0 years (95% CI: 2.7 years - not reached) in MS negative CR patients (HR = 2.46, 95% CI: 1.48–4.11, *p* < 0.001, Fig. 4A, Supplementary Fig. 3A).

The high positivity rate of MS in CR patients and its prognostic impact indicate that MS is superior to IFE in terms of sensitivity, independent on IMWG response criteria. Indeed, 78% (69/89), 50% (78/156) and 37% (49/133) of all patients with a negative IFE were still positive in MS after induction, prior to maintenance/observation and after one year of maintenance/observation, respectively (Supplementary Table 2).

To address the impact of lenalidomide maintenance in MS positive and negative CR patients, we compared CR patients in arm A of the GMMG-MM5 trial, who received lenalidomide maintenance, with CR patients in arm B without maintenance (Fig. 4B and Supplementary Fig. 3B). Lenalidomide increased PFS in both groups, MS positive (1.4 (95% CI: 0.6–3.7) years vs. 2.1 (95% CI: 1.5—not reached) years) and MS negative (not reached vs. 3.3 (95% CI: 2.3—not reached) years) patients, suggesting that even MS negative CR patients benefit from lenalidomide maintenance. Yet, likely due to the small sample size of these subgroups, results did not reach statistical significance (*p* = 0.14 and *p* = 0.06, respectively).

### Sequential testing improves the prognostic value of mass spectrometry

**Fig. 5:**
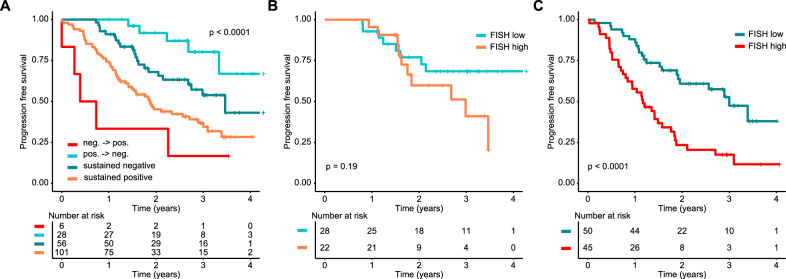
The impact of sequential mass spectrometry on PFS and the combination with the cytogenetic risk status. **A** PFS landmark analysis from 1 year (±3 months) of maintenance/observation. Patients were grouped according to the combination of the two mass spectrometry (MS) test results at the start of maintenance treatment/observation and after 1 year (±3 months) of maintenance treatment/observation. Four groups were discriminated against: sustained negativity (both MS tests negative), sustained positivity (both MS tests positive) as well as conversion from positivity to negativity (MS test positive -> negative) or vice versa (MS test negative -> positive). In (**B**) and (**C**) sustained MS negative and positive patients were further stratified by the baseline FISH risk status, respectively. High-risk cytogenetics were defined by the presence of t(4;14), t(14;16), del(17p) and/or gain(1q21).

Long-term deep responses have been associated with improved survival in MM [25, 26]. Thus, we aimed to determine the prognostic value of sequential MS testing and chose the time points prior to maintenance/observation and after 1 year. In a landmark analysis after one year of maintenance/observation, the best outcome was seen for 28 patients who converted from MS positivity to negativity (median PFS not reached, Fig. 5A). Among those, we observed a trend towards an enrichment of IgG MM patients (18% [22/125] IgG, 9% [6/66] non-IgG, *p* = 0.14), and only 7% (2/28) patients had not received lenalidomide maintenance (patients in CR in the observation arm).

The worst outcome was seen for 6 patients who converted from MS negativity to positivity with a median PFS of only 0.6 years (95% CI: 0.3 years—not reached) reflecting early disease progression. Sustained negativity (MS tests at both time points negative), which was seen in 56 patients, was associated with improved PFS (median PFS: 3.5 years, 95% CI: 2.7 years—not reached) as compared to sustained positivity (*n* = 101, median PFS: 1.9 years, 95% CI: 1.4–2.9 years; HR = 0.51, 95% CI: 0.31–0.83, *p* = 0.007). We did not observe significant OS differences between the four groups, likely due to the small number of total OS events (*n* = 20, Supplementary Fig. 4A).

When combining sequential MS with baseline cytogenetic status, there was no significant PFS difference between high and low risk patients with sustained negative MS (HR: 1.85, 95% CI: 0.73–4.73, *p* = 0.19, Fig. 5B). In contrast, high-risk status had a pronounced impact on PFS in patients with sustained MS positivity (HR: 2.85, 95%: 1.67–4.88, *p* < 0.001, Fig. 5C). For completeness, OS results are shown in Supplementary Fig. 4B, C.

### Mass spectrometry complements bone marrow minimal residual disease assessment

**Fig. 6:**
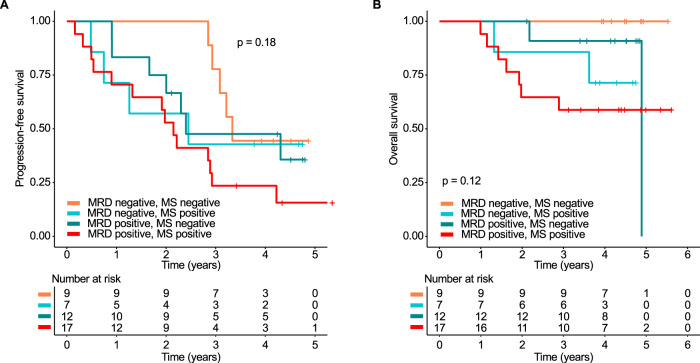
Prognostic impact of the combination of minimal residual disease and mass spectrometry. **A** PFS and (**B**) OS landmark analysis from assessment of minimal residual disease (MRD) in the bone marrow (sensitivity 10^−6^). Patients were grouped according to MRD and mass spectrometry (MS) status at this time point. Four groups were defined: MRD positive/MS positive, MRD negative/MS positive, MRD positive/MS negative, MRD negative/MS negative.

The position of MS regarding detection of MRD in the BM is one important question. MRD data were not systematically collected within the GMMG-MM5 trial. However, for 45 patients BM MRD (sensitivity 1 × 10^–6^) and MS data were available for one time point after HDM/ASCT. Details on time points of MRD evaluations are shown in Suppl. Table 3. Residual disease with at least one method, either MS or MRD, was detectable in 36 patients (80%). The worst PFS from the time point of MRD evaluation post HDM/ASCT was seen for the 17 double-positive patients (median: 2.15 years, 95% CI: 1.32 years—not reached, Fig. 6A). Yet, we did not detect a significant difference when comparing them to patients who were only positive for MRD (2.40 years, 95% CI: 2.00—not reached) or MS (2.45 years, 95% CI: 0.75-not reached) (*p* > 0.05). As expected, the best PFS was seen for the double-negative patients but we still observed disease relapses in this group (median PFS: 3.33 years, 95% CI: 3.08-not reached). We did not observe deaths in this subgroup within the observation time (Fig. 6B).

## Discussion

MS has been proposed as a minimally invasive complementary approach for monitoring of residual disease in MM patients [5]. Here we show that QIP-MS is superior to conventional response assessment in newly-diagnosed MM in terms of sensitivity and prognostic value, which is in line with recently published data of the STAMINA trial using a comparable MALDI-TOF MS approach [13]. Compared to this study, we observed a lower proportion of MS negative patients before ASCT (STAMINA: 24% vs. GMMG-MM5: 6%), which could be explained by the longer therapy prior to ASCT (up to 12 months) in the STAMINA trial. Yet, proportions of patients with negative MS after ASCT were nearly identical (STAMINA: 32% vs. GMMG-MM5: 31%), indicating that the two studies are comparable and demonstrating the feasibility of MS testing in large patient cohorts.

In both the STAMINA [13] and our own study, MS positivity had a significant negative impact on PFS even in CR patients, suggesting that positivity does not just reflect residual circulating monoclonal protein but derives from treatment-resistant tumor cells that constitute a source of disease relapse. However, we also show that the long half-life of the monoclonal protein in patients with IgG MM impacts disease monitoring, especially at early time points. We did not observe a significant difference between isotypes after one year of maintenance/observation anymore, but the optimal time point for testing still remains to be determined. In contrast to PFS, we observed only a minor or no impact on OS, probably due to limited follow-up and heterogeneous salvage therapies.

From a clinical point of view, it is an important question if QIP-MS results can be used to guide treatment decisions. While we show a significant impact of single MS testing on PFS, we demonstrate that sequential MS testing, and combination of test results with baseline biological tumor features, could strongly improve the clinical value of this technique. For instance, there were GMMG-MM5 patients who converted from MS positivity to negativity during lenalidomide maintenance. Although this subgroup showed a trend towards an enrichment of IgG MM, the excellent PFS indicates that an extended half-life of the monoclonal protein did not solely underlie this observation. Of note, the patients were not exposed to an immunomodulatory agent prior to HDM/ASCT. Thus, this subgroup most probably reflects an explicitly lenalidomide-sensitive treatment group. In contrast, patients who converted from negative to positive MS had the worst outcome, highlighting MS as a method for early detection of emerging relapse in line with other studies [11, 27]. These findings exemplify how MS testing could inform therapeutic decision making in both directions: to proceed with the current treatment if the patient becomes negative or switch to alternative treatment if the patient converts from negativity to positivity or remains MS positive, especially if the patient has high-risk status at baseline. We fully appreciate that the value of MS in therapeutic decision making needs to be addressed in the setting of prospective clinical trials.

In line with recent studies using conventional CR or MRD-negativity to define the level of response [25, 26, 28], long-term deep responses according to MS were associated with improved outcome in our study. Yet, we need to emphasize that even MS negative patients benefited from lenalidomide maintenance. A significant proportion of patients who were still negative after one year of maintenance suffered from progressive disease during the observation period of the clinical trial, indicating that QIP-MS alone is not sensitive enough to identify patients who are eligible for treatment holidays. One potential solution would be to combine MS with highly sensitive BM MRD techniques. Besides its retrospective nature, the lack of comprehensive MRD data in the GMMG-MM5 trial constitutes one of the major limitations of our study. BM MRD results were only available for a subset of patients and a comparison with MS indicates that the two approaches are complementary, which is in line with recent studies [13, 29]. In these studies, only double-positive patients had poor outcome [13, 29]. We did not detect a significant difference between double-positive patients and patients who were positive just by one technique, which could be explained by the limited patient number and follow-up. Interestingly, we observed disease progression but no deaths in double-negative patients. Disease progression in this subgroup was probably due to the limited duration of lenalidomide maintenance (maximum up to 2 years) in our trial. However, it also highlights that even a combined residual disease approach with a highly sensitive BM MRD tool was not sufficient to identify disease-free MM patients. Yet, we fully appreciate that further studies are needed to evaluate the complementary or combinatorial, and sequential value of MS testing and MRD status as well as combination with other methods, such as functional imaging. These attempts are on-going.

In conclusion, MS is a promising tool for monitoring treatment response. Recent data from the STAMINA trial [13], a further study from the MAYO clinic [7], and our own study strongly indicate that MS could replace immunofixation for the definition of CR and should be considered for IMWG response criteria. Though single MS testing should not be used to assess residual disease or prognosis, combination with baseline disease features and MRD from the BM as well as sequential MS testing improve outcome prediction. Further studies are warranted to determine the optimal time for testing and the utility of this novel approach in combination with MRD testing from the BM or imaging-based techniques and whether MS can guide therapeutic decisions.

## Supplementary information

Supplemental appendix

## Data Availability

Data from the GMMG-MM5 trial and QIP-MS samples is not publicly available. For requests, please contact the corresponding author.

## References

[1] Kumar SK, Rajkumar V, Kyle RA, van Duin M, Sonneveld P, Mateos M-V (2017). Multiple myeloma. Nat Rev Dis Prim.

[2] Pawlyn C, Morgan GJ (2017). Evolutionary biology of high-risk multiple myeloma. Nat Rev Cancer.

[3] Kumar S, Paiva B, Anderson KC, Durie B, Landgren O, Moreau P (2016). International Myeloma Working Group consensus criteria for response and minimal residual disease assessment in multiple myeloma. Lancet Oncol.

[4] Rasche L, Chavan SS, Stephens OW, Patel PH, Tytarenko R, Ashby C (2017). Spatial genomic heterogeneity in multiple myeloma revealed by multi-region sequencing. Nat Commun.

[5] Murray DL, Puig N, Kristinsson S, Usmani SZ, Dispenzieri A, Bianchi G et al. Mass spectrometry for the evaluation of monoclonal proteins in multiple myeloma and related disorders: an International Myeloma Working Group Mass Spectrometry Committee Report. Blood Cancer J. 2021; **11**. 10.1038/s41408-021-00408-4. 10.1038/s41408-021-00408-4 PMC7873248 33563895

[6] Mills JR, Kohlhagen MC, Dasari S, Vanderboom PM, Kyle RA, Katzmann JA (2016). Comprehensive assessment of M-Proteins using nanobody enrichment coupled to MALDI-TOF mass spectrometry. Clin Chem.

[7] Kohlhagen M, Dasari S, Willrich M, Hetrick M, Netzel B, Dispenzieri A (2020). Automation and validation of a MALDI-TOF MS (Mass-Fix) replacement of immunofixation electrophoresis in the clinical lab. Clin Chem Lab Med.

[8] Rögnvaldsson S, Love TJ, Thorsteinsdottir S, Reed ER, Óskarsson JÞ, Pétursdóttir Í (2021). Iceland screens, treats, or prevents multiple myeloma (iStopMM): a population-based screening study for monoclonal gammopathy of undetermined significance and randomized controlled trial of follow-up strategies. Blood Cancer J.

[9] El-Khoury H, Lee DJ, Alberge J-B, Redd R, Cea-Curry CJ, Perry J et al. Prevalence of monoclonal gammopathies and clinical outcomes in a high-risk US population screened by mass spectrometry: a prospective cohort study. SSRN Electronic J. 10.2139/ssrn.3981729. 10.1016/S2352-3026(22)00069-2 PMC9067621 35344689

[10] Campbell L, Simpson D, Ramasamy K, Sadler R (2021). Using quantitative immunoprecipitation mass spectrometry (QIP-MS) to identify low level monoclonal proteins. Clin Biochem.

[11] Santockyte R, Jin C, Pratt J, Ammar R, Desai K, Bolisetty M (2021). Sensitive multiple myeloma disease monitoring by mass spectrometry. Blood Cancer J.

[12] Kohlhagen MC, Barnidge DR, Mills JR, Stoner J, Gurtner KM, Liptac AM (2016). Screening method for M-proteins in serum using nanobody enrichment coupled to MALDI-TOF mass spectrometry. Clin Chem.

[13] Dispenzieri A, Krishnan A, Arendt B, Blackwell B, Wallace PK, Dasari S (2022). Mass-Fix better predicts for PFS and OS than standard methods among multiple myeloma patients participating on the STAMINA trial (BMT CTN 0702 /07LT). Blood Cancer J.

[14] Goldschmidt H, Mai EK, Dürig J, Scheid C, Weisel KC, Kunz C (2020). Response-adapted lenalidomide maintenance in newly diagnosed myeloma: results from the phase III GMMG-MM5 trial. Leukemia.

[15] Durie BGM, Harousseau J-L, Miguel JS, Bladé J, Barlogie B, Anderson K (2006). International uniform response criteria for multiple myeloma. Leukemia.

[16] Neben K, Lokhorst HM, Jauch A, Bertsch U, Hielscher T, van der Holt B (2012). Administration of bortezomib before and after autologous stem cell transplantation improves outcome in multiple myeloma patients with deletion 17p. Blood.

[17] Sonneveld P, Avet-Loiseau H, Lonial S, Usmani S, Siegel D, Anderson KC (2016). Treatment of multiple myeloma with high-risk cytogenetics: a consensus of the International Myeloma Working Group. Blood.

[18] Weinhold N, Salwender HJ, Cairns DA, Raab MS, Waldron G, Blau IW (2021). Chromosome 1q21 abnormalities refine outcome prediction in patients with multiple myeloma—a meta-analysis of 2,596 trial patients. Haematologica.

[19] Palumbo A, Avet-Loiseau H, Oliva S, Lokhorst HM, Goldschmidt H, Rosinol L (2015). Revised International Staging System for Multiple Myeloma: A Report From International Myeloma Working Group. J Clin Oncol.

[20] Huhn S, Weinhold N, Nickel J, Pritsch M, Hielscher T, Hummel M (2017). Circulating tumor cells as a biomarker for response to therapy in multiple myeloma patients treated within the GMMG-MM5 trial. Bone Marrow Transpl.

[21] Huhn S (2018). ELDA qASO-PCR for high sensitivity detection of tumor cells in bone marrow and peripheral blood. Methods Mol Biol.

[22] Abeykoon JP, Murray DL, Murray I, Jevremovic D, Otteson GE, Dispenzieri A (2021). Implications of detecting serum monoclonal protein by MASS-fix following stem cell transplantation in multiple myeloma. Br J Haematol.

[23] Paiva B, Puig N, Cedena M-T, Rosiñol L, Cordón L, Vidriales M-B (2020). Measurable residual disease by next-generation flow cytometry in multiple myeloma. J Clin Oncol.

[24] Rasche L, Alapat D, Kumar M, Gershner G, McDonald J, Wardell CP (2019). Combination of flow cytometry and functional imaging for monitoring of residual disease in myeloma. Leukemia.

[25] Avet-Loiseau H, San-Miguel J, Casneuf T, Iida S, Lonial S, Usmani SZ (2021). Evaluation of sustained minimal residual disease negativity with daratumumab-combination regimens in relapsed and/or refractory multiple myeloma: analysis of POLLUX and CASTOR. J Clin Oncol.

[26] Lehners N, Becker N, Benner A, Pritsch M, Löpprich M, Mai EK (2018). Analysis of long-term survival in multiple myeloma after first-line autologous stem cell transplantation: impact of clinical risk factors and sustained response. Cancer Med.

[27] Abdallah N, Murray D, Dispenzieri A, Kapoor P, Gertz MA, Lacy MQ (2022). Tracking daratumumab clearance using mass spectrometry: implications on M protein monitoring and reusing daratumumab. Leukemia.

[28] Barlogie B, Anaissie E, Haessler J, van Rhee F, Pineda-Roman M, Hollmig K (2008). Complete remission sustained 3 years from treatment initiation is a powerful surrogate for extended survival in multiple myeloma. Cancer.

[29] Eveillard M, Rustad E, Roshal M, Zhang Y, Ciardiello A, Korde N (2020). Comparison of MALDI-TOF mass spectrometry analysis of peripheral blood and bone marrow-based flow cytometry for tracking measurable residual disease in patients with multiple myeloma. Br J Haematol.

