# Supplementary material for: Implications and prognostic impact of mass spectrometry in patients with newly-diagnosed multiple myeloma
Source: Blood Cancer J. 2023 Jan 4;13(1):1. doi: 10.1038/s41408-022-00772-9 (PMC9812999; doi:10.1038/s41408-022-00772-9)
Supplement: Supplementary file 1 — Supplemental appendix [file 41408_2022_772_MOESM1_ESM.docx]

**SUPPLEMENTAL APPENDIX**

**Implications and prognostic impact of mass spectrometry in**

**patients with newly-diagnosed multiple myeloma**

Mai E. K. et al.


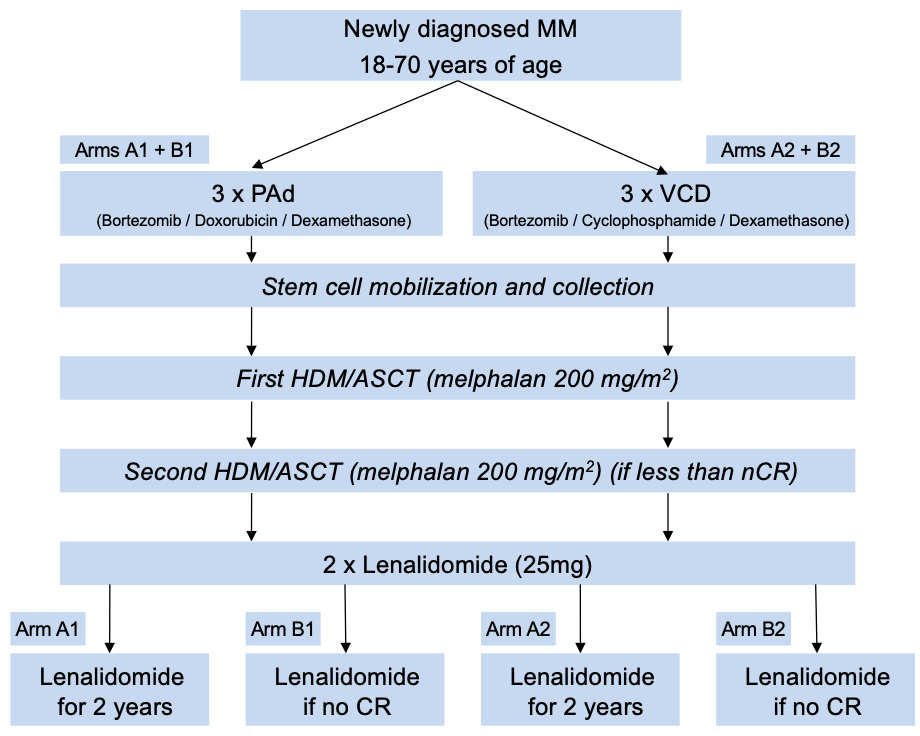
**Supplemental Figure 1: Study design and treatment within the GMMG-MM5 trial.**

Randomization was performed prior to the start of induction therapy. Maintenance therapy consisted of either Lenalidomide for 2 years or Lenalidomide in case no complete remission (CR) was achieved. Abbreviations: MM = Multiple Myeloma; GMMG = German-speaking Myeloma Multicenter Group; PAd = Bortezomib / Adriamycin / Dexamethasone; VCD = Bortezomib / Cyclophosphamide / Dexamethasone; CAD = Cyclophosphamide / Adriamycin / Dexamethasone; G-CSF = Granulocyte-Colony Stimulating Factor; HDM = high-dose melphalan; ASCT = autologous stem cell transplantation; CR = complete response; nCR = near complete remission.

**Supplemental Figure 2: Independent prognostic impact of mass spectrometry and its combination with established high-risk markers.**

Results of a multivariable model for OS from the start of maintenance therapy/observation (**A**), and after one year (±3 months) of maintenance/observation (**B**). PFS from start of maintenance therapy **(C)** and after one year of maintenance / observation **(D)** stratified by the combination of mass spectrometry (MS) and baseline high-risk cytogenetics. High risk was defined by the presence of t(4;14), t(14;16), del(17p) and/or gain(1q21).

**Supplemental Figure 3: Prognostic Impact of mass spectrometry in patients with complete response.**

(**A**) OS landmark analysis of patients in CR from the start of maintenance therapy/observation stratified by mass spectrometry (MS). In (**B**) patients were stratified by the combination of mass spectrometry and the GMMG-MM5 treatment arm (arm A = lenalidomide maintenance for two years, arm B = observation).

**Supplemental Figure 4: The impact of sequential mass spectrometry on OS and the combination with the cytogenetic risk status.**

**(A)** OS landmark analysis from 1 year (±3 months) of maintenance/observation. The patients were grouped according to the combination of the two MS test results at the start of maintenance treatment/observation and after 1 year (±3 months) of maintenance treatment/observation. We discriminated between sustained negativity (both test negative), sustained positivity (both test positive) as well as conversion from positivity to negativity (pos .> neg) or vice versa (neg -> pos). In (**B**) and (**C**) sustained negative and positive patients were further stratified by the baseline FISH risk status, respectively. High risk was defined by the presence of t(4;14), t(14;16), del(17p) and/or gain(1q21).

|  | **After induction** | | **Prior to maintenance/observation** | | **After one year of maintenance/observation** | |
| --- | --- | --- | --- | --- | --- | --- |
| **Isotype** | **MS positive** | **MS negative** | **MS positive** | **MS negative** | **MS positive** | **MS negative** |
| **IgA** | 92 (94%) | 6 (6%)* | 41 (65%) | 22 (35%) | 23 (55%) | 19 (45%) |
| **IgG** | 258 (98%) | 5 (2%)** | 143 (76%) | 45 (24%)** | 85 (59%) | 60 (41%) |
| **IgD** | 3 (75%) | 1 (25%) | 2 (100%) | 0 (0%) | 2 (67%) | 1 (33%) |
| **Bence Jones** | 65 (82%) | 14 (18%) | 24 (46%) | 28 (54%) | 17 (46%) | 20 (54%) |
| **Total** | 418 (94%) | 26 (6%) | 210 (69%) | 95 (31%) | 127 (56%) | 100 (44%) |

**Supplemental Table 1: Mass spectrometry results according to immunoglobulin isotype.**

Fisher's exact test for comparison with Bences Jones: * P<0.05; ** P<0.001. Abbreviations: MS = Mass Spectrometry; Ig = Immunoglobulin.

|  | **After induction** | | | **Prior to maintenance/observation** | | | **After one year of maintenance/observation** | | |
| --- | --- | --- | --- | --- | --- | --- | --- | --- | --- |
| **Immunofixation** | **MS positive** | **MS negative** | **Total** | **MS positive** | **MS negative** | **Total** | **MS positive** | **MS negative** | **Total** |
| **Positive** | 265 | **6** | 271 | 98 | **14** | 112 | 54 | **8** | 62 |
| **Negative** | **69** | 20 | 89 | **78** | 78 | 156 | **49** | 84 | 133 |
| **Not done** | 84 | 0 | 84 | 34 | 3 | 37 | 24 | 8 | 32 |
| **Total** | 418 | 26 | 444 | 210 | 95 | 305 | 127 | 100 | 227 |

**Supplemental Table 2: Mass spectrometry versus serum immunofixation results at three different time points.**

Discrepant results are printed in bold. Abbreviations: MS = Mass Spectrometry.

| **Patient** | **MRD** | **MS** | **Time point** | **Response** |
| --- | --- | --- | --- | --- |
| 1 | negative | negative | After consolidation | CR |
| 2 | negative | negative | After 1st ASCT | VGPR |
| 3 | negative | negative | After 2nd ASCT | CR |
| 4 | negative | negative | After 1st ASCT | CR |
| 5 | negative | negative | After 1st ASCT | CR |
| 6 | negative | negative | After 1st ASCT | VGPR |
| 7 | negative | negative | After three months of maintenance | CR |
| 8 | negative | negative | After 11 months of maintenance | CR |
| 9 | negative | positive | After three months of observation | CR |
| 10 | negative | positive | After 10 months of observation | CR |
| 11 | negative | positive | After consolidation | CR |
| 12 | negative | positive | After 2nd ASCT | VGPR |
| 13 | negative | positive | After 1st ASCT | CR |
| 14 | negative | positive | After three months of observation | CR |
| 15 | negative | positive | After three months of observation | CR |
| 16 | positive | negative | After 1st ASCT | CR |
| 17 | positive | negative | After 1st ASCT | CR |
| 18 | positive | negative | After 1st ASCT | CR |
| 19 | positive | negative | After consolidation | CR |
| 20 | positive | negative | After consolidation | CR |
| 21 | positive | negative | After consolidation | CR |
| 22 | positive | negative | After 1st ASCT | VGPR |
| 23 | positive | negative | 11 months after start of maintenance | CR |
| 24 | positive | negative | After six months of maintenance | CR |
| 25 | positive | negative | After six months of maintenance | CR |
| 26 | positive | negative | After consolidation | CR |
| 27 | positive | negative | After six months of maintenance | CR |
| 28 | positive | positive | After 1st ASCT | CR |
| 29 | positive | positive | After consolidation | CR |
| 30 | positive | positive | After four months of maintenance | CR |
| 31 | negative | negative | After three months of maintenance | CR |
| 32 | positive | positive | After consolidation | CR |
| 33 | positive | positive | After consolidation | CR |
| 34 | positive | positive | After 1st ASCT | VGPR |
| 35 | positive | positive | After 1st ASCT | CR |
| 36 | positive | positive | After consolidation | CR |
| 37 | positive | positive | After consolidation | CR |
| 38 | positive | positive | After 1st ASCT | CR |
| 39 | positive | positive | After 1st ASCT | VGPR |
| 40 | positive | positive | After consolidation | CR |
| 41 | positive | positive | After 1st ASCT | CR |
| 42 | positive | positive | After three months of maintenance | VGPR |
| 43 | positive | positive | After 1st ASCT | CR |
| 44 | positive | positive | After one month of maintenance | CR |
| 45 | positive | positive | After three months of maintenance | CR |

**Supplemental Table 3: Minimal residual disease, mass spectrometry, response status and time point of patients included in the analysis.**

Abbreviations: MRD = Minimal Residual Disease; MS = Mass Spectrometry; CR = complete response; VGPR = very good partial response; ASCT = autologous stem cell transplantation.
